# The effects of body weight loss and gain on arterial hypertension control: an observational prospective study

**DOI:** 10.1186/s40001-017-0286-5

**Published:** 2017-10-25

**Authors:** Peter Sabaka, Andrej Dukat, Jan Gajdosik, Matej Bendzala, Martin Caprnda, Fedor Simko

**Affiliations:** 1 2nd Department of Internal Medicine, Faculty of Medicine, Comenius University in Bratislava, Špitálska 24, 813 72 Bratislava, Slovak Republic; 2 Department of Pathophysiology, Faculty of Medicine, Comenius University in Bratislava, Špitálska 24, 813 72 Bratislava, Slovak Republic

**Keywords:** Obesity, Weight loss, Weight gain, Arterial hypertension

## Abstract

**Background:**

Body weight changes are associated with significant variations in blood pressure (BP). Body mass modifications may, therefore, influence hypertension control in primary care.

**Methods:**

Patients with a history of essential arterial hypertension were observed for 12 months. Anthropometric data and clinical BP were evaluated at the time of the recruitment and after 12 months of follow-up. The association between (body mass index) BMI change and BP control was analyzed by logistic regression.

**Results:**

Sixteen thousand five hundred and sixty-four patients were recruited, while 13,631 patients (6336 men; 7295 women) finished the 1-year follow-up. In obese patients, a BMI decrease by at least 1 kg/m^2^ was negatively associated with uncontrolled hypertension at the end of the follow-up (men *p* < 0.0001, OR = 0.586, 0.481–0.713, women *p* < 0.001, OR = 0.732, 0.611–0.876). A similar association was observed in overweight patients (men *p* < 0.05, OR = 0. 804, 95% CI: 0.636–0.997, women *p* < 0.05, OR = 0.730, 95% CI: 0.568–0.937). A BMI increase of at least 1 kg/m^2^ was associated with a significantly higher odd of uncontrolled hypertension in obese (men *p* < 0.001, OR = 1.471, 1.087–1.991, women *p* < 0.001, OR = 1.422, 1.104–1.833) and overweight patients (men *p* < 0.0001, OR = 1.901, 95% CI: 1.463–2.470, women *p* < 0.0001, OR = 1.647, 95% CI: 1.304–2.080).

**Conclusions:**

Weight loss is inversely associated and weight increase is positively associated with the probability of uncontrolled hypertension in obese and overweight hypertensives.

## Background

Arterial hypertension is a major global public health problem. It is estimated that more than 1 billion people suffer from arterial hypertension and its prevalence is expected to rise in the coming decades [1]. Effective blood pressure (BP) control leads to regression of blood pressure-induced organ damage and reduction of cardiovascular risk [2–5]. Despite the well-recognized negative impact of arterial hypertension on cardiovascular prognosis, its control is regarded as very poor worldwide. The proportion of arterial hypertension patients with well-controlled BP is lower than 50% in most developed countries [6]. Another serious public health problem is the rising burden of overweight and obesity prevalent in more than one-third of the world’s population [7]. In developed countries like USA, the prevalence of obesity and overweight is estimated to be as high as 66% [8].

There is a large body of evidence suggesting that the risk of developing arterial hypertension and body weight (BW) increase/obesity is mutually interdependent [9–17]. The relationship between obesity and hypertension has been described as early as in 1923 [16]. However, decisive evidence emerged much later in the Framingham Heart Study, where the risk of developing arterial hypertension was observed to be approximately two-times higher in the obese population [17]. Later, it was observed that there is a linear association between BW and BP and that every 4.5 kg increase in body weight results in an increase of 4 mmHg in systolic blood pressure (SBP) [9]. On the other hand, weight loss results in a decrease in both SBP and diastolic BP (DBP) [11, 12].

Although weight reduction is recommended in the guidelines as an essential part of non-pharmacological management of hypertension, it is often neglected by both patients and primary care physicians [18]. Thus, the relevance and effectiveness of weight reduction in the management of arterial hypertension in daily clinical practice is still obscure.

Since weight increase is associated with increase in systolic and diastolic blood pressure, we hypothesize that increase in weight may impair the effectiveness of arterial hypertension therapy and could result in higher proportion of patients with uncontrolled hypertension in primary care. We conducted prospective observational study in which the primary goal was to assess the presence of uncontrolled hypertension at the end of the 1-year follow-up period and to assess an effect of weight increase and decrease on the uncontrolled hypertension as a primary endpoint.

## Methods

We conducted partial data analysis from the MIRROR Slovakia Study, which was a nation-wide prospective observational study. The primary goal of the study was to assess the mode and efficiency of antihypertensive therapy in primary care during a 1-year follow-up of patients with known history of essential arterial hypertension. The subanalysis focused on the relationship between BMI changes and the level of hypertension control during the 1-year follow-up. Its primary goal was to assess the effect of weight increase and decrease on the uncontrolled hypertension as a primary endpoint. Patients in the MIRROR Slovakia Study were recruited proportionally from all parts of Slovakia by general practitioners and internists. They had medical histories of arterial hypertension, regardless on the niveau of hypertension control, and were older than 18 years. The exclusion criteria included a history of oncologic disease except total remission, pregnancy, thyrotoxicosis, a history of hypercorticism or therapy by corticoids, liver cirrhosis, malabsorption syndrome, and end-stage kidney disease. All patients signed an informed consent. At the recruitment and 1-year follow-up visits, the weight and height of the volunteers were obtained. Body mass index (BMI) was calculated as the BW in kilograms divided by the square of the height in meters. BP was measured by a certified automated sphygmomanometer after 5 min of rest in a sitting position on both arms three times, separated by 1 min interval. The mean value (of the three values taken) for each arm was calculated, and the higher mean value was used as definitive clinical BP. The target values for hypertension control were determined according to the 2013 European Society of Cardiology/European Society of Hypertension (ESC/ESC) guidelines [18]. Uncontrolled hypertension (used as primary endpoint for statistical analysis) was defined as clinical BP ≥ 140/90 mmHg, in patients with diabetes mellitus ≥ 140/80 mmHg and in elderly patients of over 80 years of age ≥ 150/90 mmHg. Obesity was defined as BMI ≥ 30 kg/m^2^ and overweight as BMI of 25.0–29.9 kg/m^2^. Significant weight reduction was defined as a decrease in BMI by at least 1 kg/m^2^ and significant weight increase as an increase in BMI by at least 1 kg/m^2^.

During the follow-up, the general practitioners and internists were free to change the pharmacologic antihypertensive therapy and/or recommend regime and diet changes; however they were instructed to adhere to recommendations of ESC/ESH guidelines and were also strictly instructed to provide the same level of medical attention to each patient [18]. According to observational design, investigators did not interfere with pharmacological and non-pharmacological therapy. To assess the potency of the pharmacological therapy between groups, the doses of the antihypertensive drugs were obtained by questionnaire. The doses were expressed as the proportion of doses in mg to the minimal effective doses as defined by the State Institute for Drug Control [19].

### Statistical analysis

The descriptive data were provided as mean ± standard deviation. The statistical significance of the differences in the means of quantitative variables was assessed using ANOVA with Tukey posts hoc test. Normal distribution of quantitative variables was verified using Kolmogorov–Smirnov test. Qualitative variables were compared using Chi-square test. The statistical significance of the association between BMI change and arterial hypertension control status at the end of the follow-up (uncontrolled hypertension represented the primary endpoint) was assessed using binary logistic regression analysis. Age, baseline systolic and diastolic blood pressure, creatinine, and number of minimal effective doses of antihypertensive drugs at the beginning and at the end of the follow-up were included in the analysis as possible confounders. Cohorts with no significant BMI change were used as reference groups for test purposes. Differences with* p* values of less than 0.05 were considered as statistically significant. For the statistical analysis, the SPSS version 20 of Windows was used.

The study was approved by an ethical committee of Ministry of Health of Slovak Republic.

## Results

Sixteen thousand five hundred and sixty-four patients were recruited, while 13,631 patients (6336 men; 7295 women) finished the 1-year follow-up and their data were available for analysis. Eight hundred and twenty-seven patients died and 2106 patients were lost to follow-up. At the end of the follow-up, 4162 (65.7%) men had no significant BMI change; 1489 (23.5%) had BMI decreased; and 685 (10.8%) had BMI increased and of women 4651 (63.8%) had no significant BMI change; 1634 (22.4%) had BMI decreased; and 1010 (13.8%) had the BMI decreased according to study criteria. The baseline characteristics of the cohorts are displayed in Table 1. At the baseline, there were no significant differences in patient cohorts regarding age, BMI, plasmatic creatinine concentration, and diabetes mellitus prevalence (Table 1). In systolic blood pressure and diastolic blood pressure, very small but statistically significant differences were observed (Table 1), and these variables were included in the later analysis as possible confounders.

**Table 1:** Basic cohort characteristic

	Men			Women		
	No BMI change	BMI decrease	BMI increase	No BMI change	BMI decrease	BMI increase
*N*	4162 (65.7%)	1489 (23.5%)	685 (10.8%)	4651 (63.8%)	1634 (22.4%)	1010 (13.8%)
Age	58.65 ± 11.85	58.25 ± 11.70	58.53 ± 11.87	63.57 ± 11.57	63.41 ± 11.02	36.36 ± 11.09
BMI	29.73 ± 3.89	30.17 ± 4.32	29.98 ± 4.95	29.11 ± 4.73	30.38 ± 4.71	29.78 ± 5.10
SBP (mmHg)	140.45 ± 15.99	140.51 ± 17.35	140.02 ± 16.57	140.50 ± 16.16	140.98 ± 17.42*	140.35 ± 16.21
SBP at 12 months (mmHg)	132.94 ± 10.48*	131.06 ± 9.72*	134.41 ± 11.58*	131.21 ± 9.70*	130.21 ± 10.48*	132.36 ± 11.49*
DBP (mmHg)	85.21 ± 8.74*	85.02 ± 9.88*	84.75 ± 7.94*	84.89 ± 9.74*	84.93 ± 9.81	83.75 ± 9.79*
DBP at 12 months (mmHg)	80.43 ± 6.81*	80.25 ± 6.9*	82,19 ± 7,0*	79.59 ± 6.8*	78.83 ± 6.7*	80.55 ± 8.0*
Creatinine (μmol/l)	82.63 ± 18.90	81.78 ± 18.42	82.53 ± 18.09	81.78 ± 17.96	82.83 ± 19.37	80.23 ± 17.58
Minimal effective doses of medication	3.89 ± 2.28	4.09 ± 2.35	4.04. ± 2.54	3.36 ± 2.28	3.89 ± 2.26	4.03 ± 2.43
Diabetes mellitus (%)	14.8	15.0	14.9	12.7	12.9	12.4
Controlled hypertension (%)	42.27	38.88	42.62	43.44	42.90	42.62
Smoking (%)	32.40	31.30	31.42	15.39	15.46	15.24
Anamnesis of stroke (%)	6.19	6.28	6.79	5.41	5.25	5.38
Anamnesis of MI (%)	10.88	10.73	10.62	5.50	5.01	5.78
BB (%)	31.83	29.50	29.93	35.10	34.13	32.80
ACEI/sartans (%)	89.83	90.42	89.70	92.03	93.00	91.72
Diuretics (%)	39.86	37.96	40.86	40.78	40.66	41.90
CCB (%)	35.69	37.75	34.10	34.37	33.25	34.21
CAH (%)	10.55	9.56	10.94	12.78	11.83	11.57

Values are displayed as mean ± SD

*ACEI* Angiotensin-converting enzyme inhibitors; *BMI* body mass index (kg/m^2^); *BB* beta blockers; *CAH* central antihypertensives; *CCB* calcium channel blockers; *DBP* diastolic blood pressure; *SBP* systolic blood pressure (mmHg); *MI* anamnesis of myocardial infarction

* Significantly different (*p* < 0.05) than in other cohorts by weight change using ANOVA with Tukey post hoc test for quantitative variables and Chi-square test for qualitative variables

In men, obesity and overweight were positively associated with the presence of uncontrolled hypertension in men, at the baseline. Hypertension was not sufficiently controlled in 51.2% of men with BMI < 25 kg/m^2^, 56.1% of overweight men, and 61.4% of obese men (Table 2). In women, there was also a significant difference between the proportion of uncontrolled hypertension in obese women (60.9%) and in women with BMI < 25 kg/m^2^ (54.2%) but not between overweight women and women with BMI < 25 kg/m^2^ (Table 2).

**Table 2:** Association between uncontrolled hypertension and obesity/overweight at the baseline

BMI at the baseline	*N*	Uncontrolled (%)	OR	95% CI	*p*
Men BMI 25–29.9	3029	56.1	1.174	1.016–1.356	< 0.0001
Men BMI ≥ 30	2423	61.4	1.461	1.262–1.690	< 0.0001
Women BMI 25–29.9	2988	56.0	1.076	0.9402–1.231	0.288
Women BMI ≥ 30	3095	60.9	1.321	1.151–1.515	< 0.0001

*BMI* body mass index (kg/m^2^); *CI* confidence interval; *OR* odds ratio of the association between obesity/overweight and uncontrolled hypertension compared to subgroups of men and women with BMI less than 25 kg/m^2^; *P* probability (association of uncontrolled hypertension and obesity and overweight at the baseline, compared to subgroups of men and women with BMI less than 25 kg/m^2^ cohorts, compared using Chi-square test)

### BMI decrease and hypertension control

Logistic regression show that in the cohorts of obese and overweight men and women at the baseline, BMI decrease during the follow-up by at least 1 kg/m^2^ was inversely associated with the presence of uncontrolled hypertension at the end of the follow-up. The logistic regression used baseline age, systolic blood pressure (SBP), diastolic blood pressure (DBP), creatinine, and doses of antihypertensive therapy at the beginning and at the end of the follow-up as possible confounding variables (Table 3). Weight loss surrogated by BMI decrease resulted in a lower risk of suffering from uncontrolled hypertension in obese and overweight patients, with the strongest association observed in the cohort of obese men, followed by that of obese women. The BMI decrease had no significant effect on AH control in men and women with BMI less than 25 kg/m^2^ (Table 3).

**Table 3:** Association between BMI decrease by at least 1 kg/m^2^ during the 1-year follow-up and the risk of uncontrolled hypertension at the end of the follow-up by logistic regression

BMI at the baseline	Gender	*p*	OR	95% CI
BMI < 25	Men	0.397	0.771	0.423–1.406
	Women	0.242	0.743	0.451–1.223
BMI 25–29.9	Men	< 0.05	0.804	0.636–0.997
	Women	< 0.05	0.732	0.568–0.937
BMI ≥ 30	Men	< 0.0001	0.586	0.481–0.713
	Women	< 0.001	0.730	0.611–0.876

*BMI* body mass index (kg/m^2^); *CI* confidence interval; *OR* odds ratio of the association between BMI decrease and uncontrolled hypertension at the end of the follow-up (compared to cohorts with no significant BMI change); *P* probability (association assessed using binary logistic regression, age, baseline systolic and diastolic blood pressure, creatinine, and number of minimal effective doses of antihypertensive drugs at the beginning and at the end of the follow-up were included in the analysis as possible confounders)

### BMI increase and hypertension control

Logistic regression analysis shows that in the cohorts of men and women with obesity and overweight, BMI increase by at least 1 kg/m^2^ during the follow-up was positively associated with uncontrolled hypertension at the end of the follow-up. The logistic regression used baseline age, SBP, DBP, creatinine, and doses of antihypertensive therapy at the beginning and at the end of the follow-up as possible confounding variables (Table 4). In the cohorts of men and women with BMI < 25 kg/m^2^, a slight trend was observed, but association was not statistically significant (Table 4). The highest odds ratio was observed in the cohort of overweight men, followed by that of overweight women (Table 4).

**Table 4:** Association between BMI increase by at least 1 kg/m^2^ during the 1-year follow-up and the risk of uncontrolled hypertension at the end of the follow-up by logistic regression

BMI at the baseline	Gender	*p*	OR	95% CI
BMI < 25	Men	0.131	1.383	0. 908–2.104
	Women	0.247	1.166	0.846–1.607
BMI 25–29.9	Men	< 0.0001	1.901	1.463–2.470
	Women	< 0.0001	1.647	1.304–2.080
BMI ≥ 30	Men	< 0.001	1.471	1.087–1.991
	Women	< 0.001	1.422	1.104–1.833

*BMI* body mass index (kg/m^2^); *CI* confidence interval; *OR* odds ratio of the association between BMI increase and uncontrolled hypertension at the end of the follow-up (compared to cohorts with no significant BMI change); *P* probability (association assessed using binary logistic regression, age, baseline systolic and diastolic blood pressure, creatinine, and number of minimal effective doses of antihypertensive drugs at the beginning and at the end of the follow-up were included in the analysis as possible confounders)

## Discussion

This prospective follow-up of patients in primary care for a duration of 1 year has revealed that changes in the body weight in terms of BMI variations above 1 kg/m^2^ influence the effectiveness of antihypertensive therapy. There is a large body of evidence linking weight changes to changes in BP. However, no comprehensive study has been conducted before now to explore this link, and the role of weight management in the complex antihypertensive therapy in primary care is yet to be determined. Our results emphasize the significance of BW control in improving the efficacy of antihypertensive treatment in primary care and highlights not just the benefits of weight reduction but also the benefits of, at least, maintaining a stable weight over weight gain.

### Weight loss and hypertension control

In our study, in obese men and women, BMI decrease during the follow-up by at least 1 kg/m^2^ was inversely associated with uncontrolled hypertension at the end of the follow-up. The odds ratio for uncontrolled hypertension was lower in obese men than in obese women, indicating a stronger association in men. This association was observed also in the groups of overweight men and women, but the odds ratios were higher in patients with obesity. In men and women with BMI less than 25 kg/m^2^, no effect on AH control has been observed. Thus, weight reduction in obese patients seems to bring a greater benefit in terms of achieving hypertension control compared to overweight patients and is of greatest importance in obese men. Previous studies indicate an association between weight loss and reduction of SBP and DBP. A meta-analysis of 25 studies found a linear relationship between weight loss and blood pressure and showed that the decrease in weight by 1 kg is associated with approximately 1 mmHg decline in SBP [12]. A large prospective study (The Trial of Hypertension Prevention) with more than 500 prehypertensive individuals assigned to weight loss programme showed not just a significant decrease in SBP and DBP but also a significantly lower proportion of hypertensive patients in the treatment arm at the end of the follow-up [14].

### Weight increase and hypertension control

BMI increase was positively associated with poor hypertension control at the end of the follow-up in obese and overweight patients. In overweight men and women, higher odds ratios were observed, indicating a stronger association than in obese individuals. The effect of weight increase on BP and the linear association between weight increase and SBP have been described [9]. In a more recent study, weight gain during 1-year follow-up was associated with an increase in SBP and DBP in young adults regardless of baseline BMI [11]. On the basis of our findings, it can be supposed that even maintenance of a stable weight is beneficial compared to weight gain in terms of appropriate hypertension control. Since obesity is associated also with other negative metabolic effects, like the atherogenic changes in fasting and postprandial lipoprotein profile, weight management should be a crucial part of the complex treatment of patients with arterial hypertension [20].

### Pathophysiologic background

The potential pathophysiologic implications of adiposity on blood pressure increase and poor BP control have been identified. Most of them are linked to water and salt metabolism and regulation of sodium excretion. Obesity leads to the up-regulation of renin–angiotensin–aldosterone axis and sodium and fluid retention [21, 22]. Moreover, leptin, the hormone produced by adipose tissue, is excessively secreted in obesity. This adipokine stimulates the sympathetic sensitivity of the kidney, which may lead to excessive sodium and fluid retention [23]. This effect is probably aldosterone dependent [24]. Furthermore, insulin resistance with hyperinsulinemia induced by abdominal obesity may attenuate renal sodium excretion [25, 26]. On the other hand, the concentration of ghrelin, the hormone produced by the gastric mucosa during fasting period that stimulates the excretion of sodium, is lowered in obese patients and rises during weight loss. In animals, ghrelin increase results in the reduction of BP [27]. Increase in ghrelin concentration stimulated by weight loss might contribute to the improvement of BP control. Additionally, obesity is associated with histologic and macroscopic kidney abnormalities, which may alter the kidney competence to maintain sodium and fluid homeostasis. However, it is supposed that these renal alterations are reversible and could be improved by weight loss [28].

### Limitations of the study

Participants of our study did not undergo a controlled weight reduction programme, since the study was only observational in nature. Therefore, it was not possible to quantify the participation of patients in these non-pharmacologic strategies, such as diet, salt intake, and regular physical activity changes. To minimize this effect, general practitioners were instructed to educate patients about non-pharmacologic means of blood pressure management equally.

In order to minimize the effect of comorbidities that can lead to undesirable loss of weight, the patients with known history of oncologic disease (except in the case of total remission), pregnancy, thyrotoxicosis, liver cirrhosis, malabsorption syndrome, and end-stage kidney disease were excluded. However, there was a possibility that in some patients, these conditions might develop during the follow-up. Also, the possibility of significant worsening of glomerular filtration rate during the follow-up was not ruled out.

Patients in our study differ in terms of antihypertensive therapy. Moreover, the pharmacological therapy was modified in a substantial number of patients during the follow-up. To decrease the effect of possible differences in pharmacological therapy as a confounding factor, the doses of antihypertensive therapies administered at the baseline and at the end of the follow-up were included in the logistic regression analysis as a possible confounder. All the patients were followed up on a regular basis of 3 months, and the general practitioners were strictly instructed to provide the same level of medical attention to each patient. However, there is a possibility of difference in the frequency of therapy modification.

Also, the duration of the follow-up period should be considered. Our patients were observed for the period of 1 year; thus, it was not possible to assess the long-term effect of weight loss. A previous study on obese patients who underwent bariatric procedure has shown that BP increased again on pre-surgery values during 8-year follow-up [29]. However, another study not using bariatric surgery has shown that the effect of weight loss lasts for 3 years minimally if the weight remains at least 4% lower than the baseline weight [14].

Most previous studies used body weight change as an indicator of weight loss. We used delta BMI rather than delta body weight, because BMI is a function of body adiposity [30].

## Conclusion

In our large observational prospective study, it has been shown that BW reduction by at least 1 kg/m^2^ was associated with lower risk of uncontrolled hypertension at the end of 1-year follow-up in patients with obesity and overweight. This emphasizes the importance of BW control in the management of cardiovascular risk in patients with arterial hypertension, and it should be considered as an unavoidable recommendation in the complex treatment of arterial hypertension in obese and overweight individuals. On the other hand, weight increase by at least 1 kg/m^2^ during 1-year follow-up enhanced the risk of uncontrolled hypertension compared to maintaining approximately the same weight. It is suggested that weight loss or at least stabilization of BW may improve the control of hypertension in obese hypertonics and in overweight hypertensive patients.

## Abbreviations

BMI: body mass index
BP: blood pressure
BW: body weight
DBP: diastolic blood pressure
SBP: systolic blood pressure
OR: odds ratio
RR: relative risk
